# Multidimensional fetal flow imaging with cardiovascular magnetic resonance: a feasibility study

**DOI:** 10.1186/s12968-018-0498-z

**Published:** 2018-11-29

**Authors:** Datta Singh Goolaub, Christopher W. Roy, Eric Schrauben, Dafna Sussman, Davide Marini, Mike Seed, Christopher K. Macgowan

**Affiliations:** 1 Medical Biophysics, University of Toronto, Toronto, ON Canada; 2 Translational Medicine, Hospital for Sick Children, Toronto, ON Canada; 3 Electrical, Computer, and Biomedical Engineering, Ryerson University, Toronto, ON Canada; 4 Institute for Biomedical Engineering, Science and Technology (iBEST), Ryerson University and St. Michael’s Hospital, Toronto, ON Canada; 5 Division of Pediatric Cardiology, Hospital for Sick Children, Toronto, ON Canada; 6 Paediatrics, University of Toronto, Toronto, ON Canada

**Keywords:** Fetal, Golden angle radial, Motion correction, Retrospective gating, Compressed sensing, Phase contrast MRI

## Abstract

**Purpose:**

To image multidimensional flow in fetuses using golden-angle radial phase contrast cardiovascular magnetic resonance (PC-CMR) with motion correction and retrospective gating.

**Methods:**

A novel PC-CMR method was developed using an ungated golden-angle radial acquisition with continuously incremented velocity encoding. Healthy subjects (*n* = 5, 27 ± 3 years, males) and pregnant females (n = 5, 34 ± 2 weeks gestation) were imaged at 3 T using the proposed sequence. Real-time reconstructions were first performed for retrospective motion correction and cardiac gating (using metric optimized gating, MOG). CINE reconstructions of multidimensional flow were then performed using the corrected and gated data.

**Results:**

In adults, flows obtained using the proposed method agreed strongly with those obtained using a conventionally gated Cartesian acquisition. Across the five adults, bias and limits of agreement were − 1.0 cm/s and [− 5.1, 3.2] cm/s for mean velocities and − 1.1 cm/s and [− 6.5, 4.3] cm/s for peak velocities. Temporal correlation between corresponding waveforms was also high (R~ 0.98). Calculated timing errors between MOG and pulse-gating RR intervals were low (~ 20 ms). First insights into multidimensional fetal blood flows were achieved. Inter-subject consistency in fetal descending aortic flows (*n* = 3) was strong with an average velocity of 27.1 ± 0.4 cm/s, peak systolic velocity of 70.0 ± 1.8 cm/s and an intra-class correlation coefficient of 0.95 between the velocity waveforms. In one fetal case, high flow waveform reproducibility was demonstrated in the ascending aorta (*R* = 0.97) and main pulmonary artery (*R* = 0.99).

**Conclusion:**

Multidimensional PC-CMR of fetal flow was developed and validated, incorporating retrospective motion compensation and cardiac gating. Using this method, the first quantification and visualization of multidimensional fetal blood flow was achieved using CMR.

**Electronic supplementary material:**

The online version of this article (10.1186/s12968-018-0498-z) contains supplementary material, which is available to authorized users.

## Introduction

Disruption of the fetal circulation by congenital heart disease (CHD) can result in injury to critical organs and possible fetal death [1]. In such cases, premature delivery with subsequent intervention may be recommended, but this option must be weighed against the risks of prematurity such as infection, impaired organ development and cognitive delay. Emerging treatments intended to improve the fetal circulation include drugs provided to the fetus through the maternal circulation, maternal oxygen supplementation, and percutaneous surgical correction of fetal cardiovascular anatomy [2–4]. Selecting the most appropriate therapy and monitoring its efficacy, however, requires an accurate assessment of the fetal circulation.

Fetal cardiovascular magnetic resonance (CMR) methods have recently been developed to assess fetal anatomy and blood flow in late gestation using steady state free precession and flow sensitive phase contrast CMR (PC-CMR). Using these methods, fetal blood flow and oxygen delivery have been measured in a variety of fetal CHD including left-heart disease and transposition of the great arteries [5, 6]. However, quantification of fetal flow by PC-CMR remains challenging. The primary hurdles are:

1. Fetal vascular and cardiac structures are small with complex morphology. To quantify flow accurately in the heart and major vessels, a high spatial resolution (≤ 1 × 1 mm^2^) is beneficial.
2. Fetal heart rates are high (~ 110–180 bpm), requiring acquisitions at high temporal resolution. Based on adult studies using PC-CMR, a minimum of 15 cardiac phases are required to resolve blood flow, which corresponds to a temporal resolution of approximately 30 ms in fetal applications [7].
3. Motion from maternal respiration or gross fetal movements will corrupt portions of the data. Thus, the fetal PC-CMR acquisition and analysis must monitor and correct for these motions.
4. Conventional electrocardiogram (ECG) or peripheral pulse gating methods cannot be used to synchronize fetal data acquisition and reconstruction [8]. Thus, a method for extracting this signal non-invasively is needed.

Previous PC-CMR studies of fetal blood flow have included Cartesian sampling with retrospective gating [9–11]. However, these were sensitive only to flow through the prescribed slice and did not correct for motion, often requiring repeat acquisitions to avoid image artifacts. Here, we propose, validate, and demonstrate the feasibility of a novel PC-CMR approach for measuring complex fetal hemodynamics that compensates for motion while meeting the spatiotemporal demands of fetal applications. This builds on previous work using radial golden-angle sampling with compressed sensing (CS) for fetal CMR [12, 13]. A strength of the golden-angle approach is its ability to provide real-time reconstructions for motion compensation and retrospective gating [12, 13]. We adopt a similar approach but incorporate multi-dimensional flow encoding for a single-slice acquisition, hereafter referred to as “single slice vector flow”. By acquiring all three velocity components, complex flows (such as those passing through the fetal heart) may be visualized and measured. The theory behind the sampling and reconstruction of such data is described in the next section, followed by an experimental validation of the method in a flow phantom and 5 healthy adult subjects. Finally, feasibility of the method is demonstrated in scans of complex blood flow through the fetal heart and major fetal vessels of 5 healthy pregnancies.

## Theory

### Acquisition

Golden-angle radial acquisitions provide reasonably uniform k-space coverage over flexible temporal windows, which enables image reconstruction at temporal resolutions appropriate for motion correction and image-based gating [14]. Extending this approach to multi-dimensional PC-CMR, however, can dramatically reduce its temporal resolution. This limitation arises from having each direction of flow encoding acquired consecutively for a given radial spoke angle, such that the temporal resolution of the real-time reconstruction is four-fold lower than that of a similar anatomical scan. Using N_s_ spokes to reconstruct each real-time frame, the temporal resolution is 4·N_s_·TR. For typical parameters (Ns~ 4, TR~ 7 ms), this leads to a temporal resolution of 112 ms, which is too long for fetal image-based gating.

**Fig. 1:**
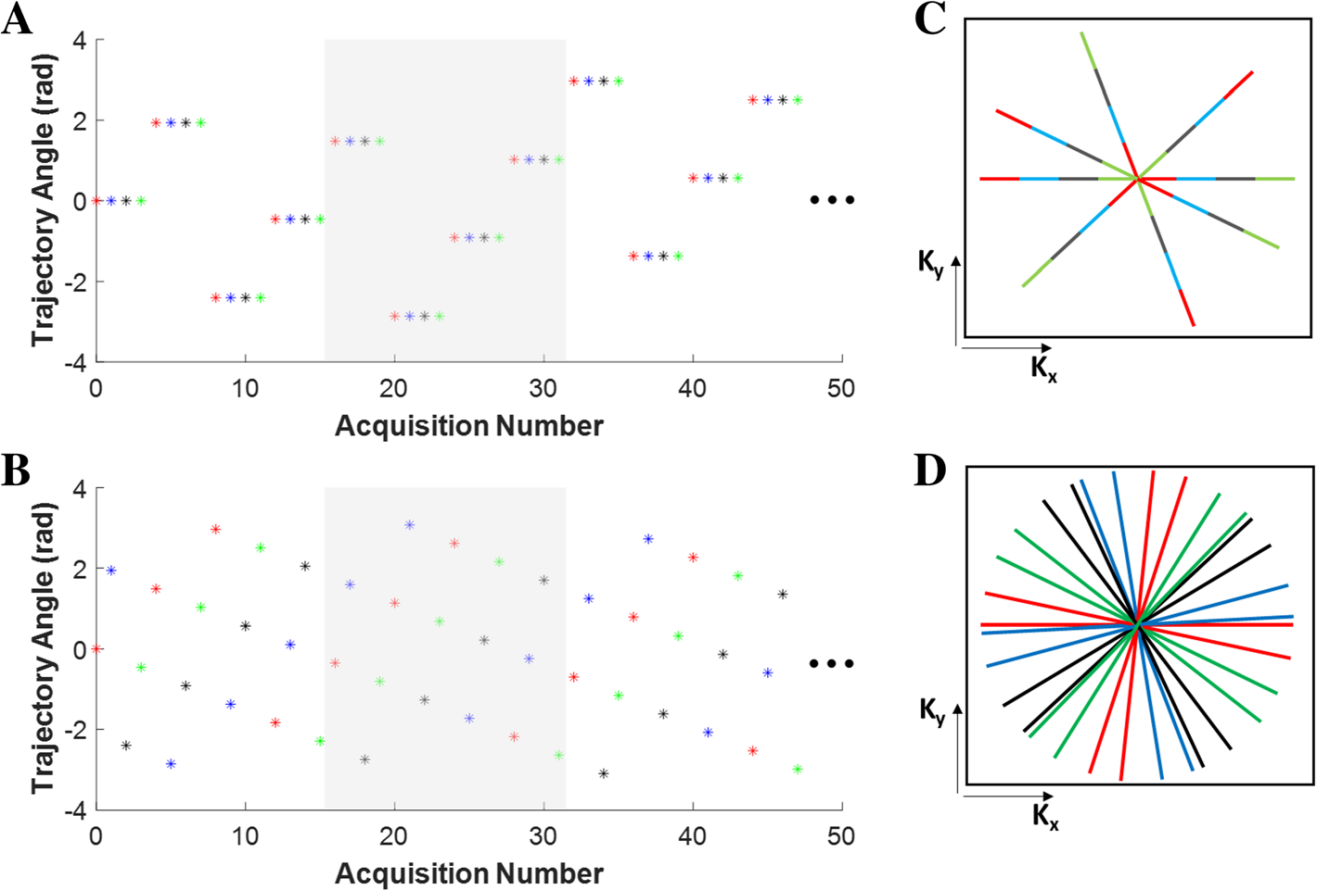
Radial sampling strategies for multidimensional flow encoding. (**a**) Conventional phase contrast  cardiovascular magnetic resonance (PC-CMR) sampling involves reacquisition of each flow encode before incrementing the trajectory angle. Velocity encodes are color coded as: flow compensation = red; through-plane encode = blue; orthogonal in-plane encodes = black and green. (**b**) The proposed novel sampling pattern synchronously increments the radial trajectory (by golden angle) and the velocity encoding direction. In (**a**, **b**), the grey overlays show the window at a fixed temporal resolution for real-time reconstructions, and the corresponding k-space coverages are shown in (**c**, **d**)

To overcome this limitation, we propose a PC-CMR acquisition that interleaves the velocity encoding directions while continuously advancing the radial trajectory by the golden angle. This approach enables reconstruction of anatomical real-time frames with an improved temporal resolution of N_s_·TR. The gain of this approach is that, for a given temporal resolution, the k-space coverage for each real-time frame is increased by a factor of four as compared to the conventional approach, as illustrated in Fig. 1.

### Reconstruction

**Fig. 2:**
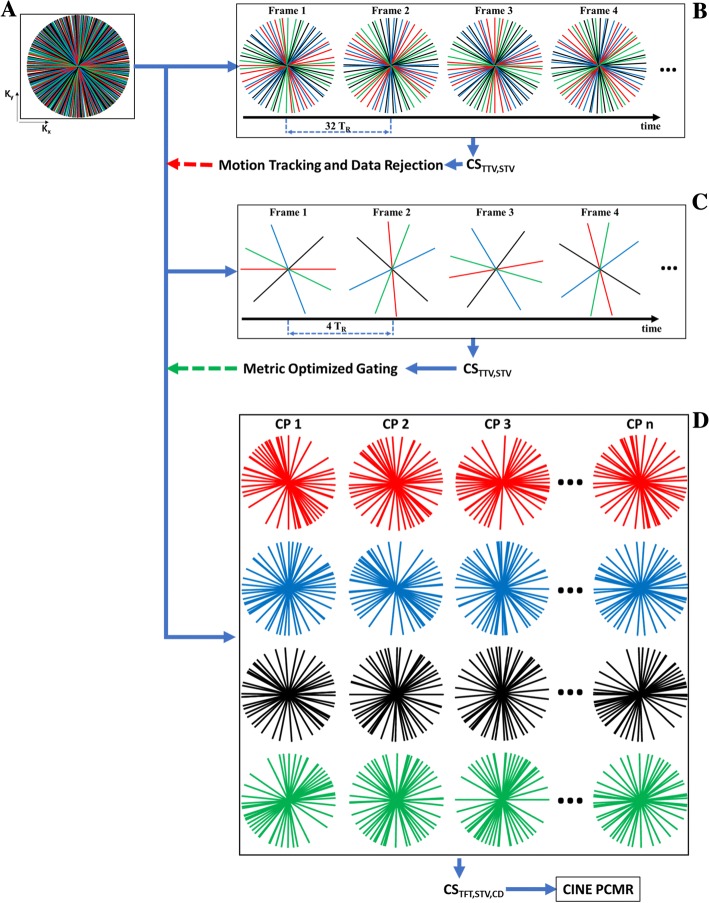
Pipeline to reconstruct fetal multidimensional PC-CMR data. (**a**) PC-CMR data is acquired with continuously incremented velocity encoding (encodes are color coded as: flow compensation = red; through-plane encode = blue; orthogonal in-plane encodes = black and green) and golden angle radial sampling. (**b**) Temporal windows of 200 ms are created for real-time reconstruction using compressed sensing (CS) with temporal and spatial total variation sparsity constraints (TTV and STV, respectively). Motion tracking and data rejection are performed, and the retained k-space data is motion corrected (red dotted arrow). (**c**) Temporal windows of 25 ms are created for real-time reconstruction using CS, again with TTV and STV constraints, and metric-optimized gating is performed on the magnitude data (green dotted arrow). (**d**) The data are binned into *n* cardiac phases (CP), and CS is used to create a multidimensional fetal flow CINE, with sparsity constraints in the STV, complex difference (CD) and temporal Fourier transform (TFT) domains

The proposed reconstruction pipeline is composed of three stages, as illustrated in Fig. 2.

#### Stage 1: Motion detection and correction

The first stage involves reconstruction of a real-time image series for retrospective detection and compensation of motion (Fig. 2b). For this purpose, we have found a real-time temporal resolution of approximately 200 ms is adequate for motion tracking. The reconstruction of such real-time frames is performed using CS, exploiting sparsity in the spatial and temporal total variation domains [15, 16]. CS is used here to remove undersampling artifacts that could impair motion detection. Data acquired during periods of through-plane motion (typically gross fetal movements) are discarded. This process uses a template matching approach, as follows. First, a reference frame is defined as the real-time frame with the highest mutual information with all other frames. Next, a region of interest (ROI) is placed within that reference frame around a target structure (e.g. fetal chest), and all other frames are registered to this ROI by rigid translation. The average mutual information between a frame and all others is then calculated over the ROI. This is repeated for each frame to identify outliers (frames with an average mutual information less than the mean of the distribution by 1.5× the interquartile range). These frames are deemed to be corrupted by through-plane motion and thus discarded from further analysis.

The remaining data are then corrected for in-plane displacements (typically maternal respiration), based on the ROI translations measured in the previous section. These ROI translations are interpolated to the repetition time (TR) of the original PC-CMR acquisition and used to motion-correct each spoke of the retained raw data (by applying the inverse phase offset corresponding to each translation) [12]. In the proposed approach, only translational motions of fetal structures are considered since these are attributed to maternal respiration. Other types of motion (such as rotation, shear or dilations) of fetal structures are not considered since they are associated with gross fetal movements which include components of through-plane displacement (and are thus excluded from the reconstruction using the data rejection scheme described above).

#### Stage 2: Fetal cardiac gating

The second stage of the reconstruction involves retrospective detection of the fetal cardiac cycle (RR intervals) for subsequent grouping of data into cardiac phases. This involves a second real-time reconstruction, but at a higher temporal resolution for detection of high fetal heart rates. Using the motion compensated data from the previous stage, images are reconstructed at an effective temporal resolution of 25 ms (Fig. 2c). This results in high radial undersampling (*R* = 100), but which can again be reconstructed with CS using spatial total variation and temporal total variation sparsity.

To determine the RR intervals, a retrospective gating approach is performed using an iterative multiparameter metric-optimized gating (MOG) strategy [17]. This approach sorts real-time images into combined cardiac frames until entropy is minimized within an ROI encompassing the periodic phenomenon of interest (such as fetal myocardial motion or in-flow effects in blood vessels). In this study, a spatiotemporal measure of entropy (E) is minimized, as defined by the following expression:

$$ E={\sum}_t{\sum}_x{\sum}_y\frac{S_{x,y,t}}{B}\log \left(\frac{S_{x,y,t}}{B}\right) $$

where *S* is the absolute pixel value at temporal index *t* and spatial position (*x*, *y*) in the ROI and *B* is a normalizing term given by ${\left[{\sum}_t{\sum}_x{\sum}_y{S}_{x,y,t}^2\right]}^{\frac{1}{2}}$.

#### Stage 3: CINE reconstruction

In this final stage, the calculated RR intervals are used to sort the motion compensated k-space data into cardiac phases, and a high-quality CINE PC-CMR reconstruction is performed (Fig. 2d). The CINE reconstruction uses CS with sparsity in the spatial total variation, temporal Fourier transform and complex difference domains. Images with periodic phenomena are sparse in the temporal Fourier domain, and flow-encoded images are sparse in the complex difference domain [18, 19]. These approaches have been previously shown to reconstruct cardiac time series images [17] and to quantify blood flow from highly undersampled data by Kwak et al. [19]. Three separate CINE reconstructions are performed, corresponding to velocities *x, y* and *z* dimensions. Sparsity in reconstruction is minimized as follows. The complex difference is computed by performing a framewise-subtraction of the flow compensated reconstruction from the flow encoded reconstruction. The temporal Fourier and spatial total variation transforms are applied to the flow encoded and flow compensation reconstructions separately. As is typical of CS, consistency of the reconstruction is maintained by minimising the error between the multichannel data and the coil-sensitivity weighted, non-uniform fast Fourier transform of the reconstruction.

## Methods

All experiments were performed using a commercial 3 T CMR system (Prisma^FIT^, Siemens Healthineers, Erlangen, Germany). The radial PC-CMR approach described in the previous section was first validated in a phantom experiment and 5 healthy adult subjects. Its feasibility was then demonstrated in 5 healthy pregnancies. Acquisitions were performed using a gradient recalled echo (GRE) sequence with a multi-channel receiver coil. All acquisitions were performed as part of an ethically approved study, and informed consent was obtained from all subjects. All reconstructions were performed in MATLAB (MathWorks, Natick, Massachusetts, USA), modifying code originally developed by Lustig et al. and Otazo et al., and using conjugate gradient descent for optimization during CS reconstruction [15, 16]. Computer specifications were: RAM 32GB, processor Intel® Core™ i7–6700 (3.40 GHz, 8 cores). NUFFT [20] was performed on a GPU (Nvidia Geforce GTX 960, 2GB and 1024 CUDA cores). Motion compensation was based on the normalized mutual information, calculated with elastix (Image Sciences Institute, University Medical Center Ultrecht, The Netherlands) [21].

### PC-CMR acquisition protocol – Phantom

To validate the radial PC-CMR sequence, flow was measured in a phantom consisting of a computer-controlled pump connected to coiled tubing (10 mm diameter) positioned within the scanner. Flows measured using the proposed sampling strategy were compared to reference flows obtained using a conventional multi-dimensional flow encoded Cartesian PC-CMR sequence. Measurements were made along a double oblique plane to obtain velocity components along all three spatial dimensions. Constant flows (ranging from 10 to 60 mL/s) were measured in eight sections of the coiled tubing. Agreements between the radial and Cartesian measurements for the mean and peak spatial velocities across the cross-section of the tubing were quantified through linear regression for each velocity encoding dimension. Comparisons between radial and Cartesian PC-CMR measurements were made instead of validation against programmed flow for direct comparison of complex multidimensional flow vectors.

### PC-CMR acquisition protocol – Adult

For validating MOG, flows were measured in healthy adults (25–30 years) using the proposed method. In three subjects, acquisitions were performed axially at the level of the bifurcation of the main pulmonary artery (MPA). In the remaining two subjects, a four-chamber view of the heart was acquired. Relevant imaging parameters are summarized in Table 1.

**Table 1:** PCMR imaging parameters

Parameters	Adult Acquisition	Fetal Acquisition
Subjects	A1, A2, A3, A4, A5	P1, P2, P3, P4, P5
Flip Angle	15^o^	15^o^
Field of View (mm^2^)	256 × 256	256 × 256
Resolution (mm^3^)	1 × 1 × 4	1 × 1 × 4
VENC (cm/s)	150	80 (P1–3) 150 (P4–5)
Number of Flow Encodes	4	4
Spokes per Slice	4000 (1000 per encode)	3600–4800 (900–1200 per flow encode)
TR / TE (ms)	6.5 / 4.0	6.5 / 4.0
Scan Length (s)	26	22–28

During acquisition of the radial PC-CMR data, pulse gating waveforms were recorded for later comparison with MOG-based gating. Two CINEs were reconstructed from the radial data (one with MOG and the other with pulse gating) and measured velocities were compared. To test reproducibility of the flow measurements and gating performance, scans were repeated three times consecutively in each subject.

### PC-CMR acquisition protocol – Fetal

Five healthy pregnant women (subject identifiers P1-P5; gestational ages 32–36 weeks) were recruited and scanned under free breathing conditions using the proposed PC-CMR approach. In three subjects, four-chamber views of the fetal heart were prescribed (subjects P1, P4 and P5). In the two other subjects, standard three vessel views of the fetus were prescribed (subjects P2 and P3). The imaging parameters are summarized in Table 1.

### Data analysis – Adults

Adult flow data, acquired using the radial PC-CMR acquisition, were reconstructed using the pipeline described in the Theory section and depicted in Fig. 2, but without the need for gross motion correction (Fig. 2b).

First, real-time series with four consecutive spokes per frame were reconstructed using regularization weights of 0.05 and 0.001 for temporal and spatial total variation respectively (temporal resolution of 26 ms; acceleration of 100, Fig. 2c). These images were used to detect the RR intervals for each acquisition, using MOG with an ROI placed over the heart and surrounding vasculature. Based on these RR intervals, retrospective CINE reconstructions were performed using CS (regularization weights: 0.01 for spatial total variation, 0.01 for complex difference and 0.05 for temporal Fourier transform) with the minimisation procedure alternating between flow compensation and flow encoding (Fig. 2d). This resulted in single slice vector flow measurements that were then corrected for background phase [22].

The accuracy of the MOG-based reconstruction of radial PC-CMR data was evaluated based on two quantities. First, the retrospective gating signal obtained by MOG was compared with that from the recorded pulse gating log. Specifically, the timing error was defined as the standard deviation of the differences between the corresponding RR intervals from each method. Second, quantitative evaluation of the proposed sampling scheme was performed by running Wilcoxon signed rank test on and comparing Bland-Altman plots of the mean and the peak velocities between reconstructions from the MOG and the logged pulse-gated reconstructions. Agreement between the reconstructions was analyzed by looking at the interclass Pearson correlation coefficients (R) between the flow curves.

### Data analysis – Fetal

Real-time frames for motion compensation were reconstructed using 32 consecutive spokes (temporal resolution of 208 ms; acceleration of 12) with CS (regularization weights: 0.05 for temporal total variation and 0.001 for spatial total variation). Motion compensation was performed on these real-time frames as described in the Theory. A second real-time reconstruction was then performed for gating, based on 4 consecutive spokes (temporal resolution of 26 ms; acceleration of 100) with CS (regularization weights: 0.05 for temporal total variation and 0.001 for spatial total variation). MOG was performed and the data was binned into 15 cardiac phases. The acceleration factor ranged between 5 and 12 in these final reconstructions, depending on the amount of data rejection due to through-plane motion. CINE reconstructions were performed using CS (regularization weights: 0.0025 for spatial total variation, 0.0025 for complex difference and 0.025 for temporal Fourier transform) with the minimisation procedure alternating between the flow compensation and flow encoding.

With the current pipeline, real-time reconstructions used for fetal motion compensation required approximately 5 min/slice and those used for gating required approximately 45 min/slice. Both reconstructions ran for 50 iterations. Image registration and MOG analysis each required approximately 10 min/slice. CINE reconstructions lasted approximately 30 min/slice with 80 iterations. Accounting for 2 min for manual ROI selections, the total processing time with this pipeline was approximately 102 min per slice.

### Consistency in fetal reconstructions

As a simple test of the reproducibility of this acquisition and reconstruction, fetal data were acquired twice consecutively from one participant (P3). After reconstruction, pulsatile flows though the fetal aorta were measured. The consistency of these measurements was quantified based on the intraclass correlation coefficient (ICC) and the peak velocity error between the two flow curves.

## Results

### PC-CMR validation – Phantom

Flows measured using the proposed radial PC-CMR sampling strategy showed excellent agreement with those measured using conventional Cartesian PC-CMR sampling. Along each flow dimension, linear regression analysis between the mean and peak velocities (cm/s) showed high correlation and near unitary slopes:

$$ {RV}_{mean}=\left[\begin{array}{c}0.97\\ {}1.00\\ {}0.96\end{array}\right]{CV}_{mean}+\left[\begin{array}{c}1.23\\ {}1.03\mathrm{x}{10}^{-15}\\ {}1.61\end{array}\right]\kern2em {r}^2=1.00 $$

$$ {RV}_{peak}=\left[\begin{array}{c}0.97\\ {}0.93\\ {}0.95\end{array}\right]{CV}_{peak}+\left[\begin{array}{c}1.40\\ {}1.67\\ {}1.76\end{array}\right]\kern1.6em {r}^2=\left[\begin{array}{c}0.98\\ {}0.93\\ {}0.97\end{array}\right] $$

where RV and CV are the velocities obtained from the radial and Cartesian acquisitions, respectively.

### PC-CMR validation – Adult

**Fig. 3:**
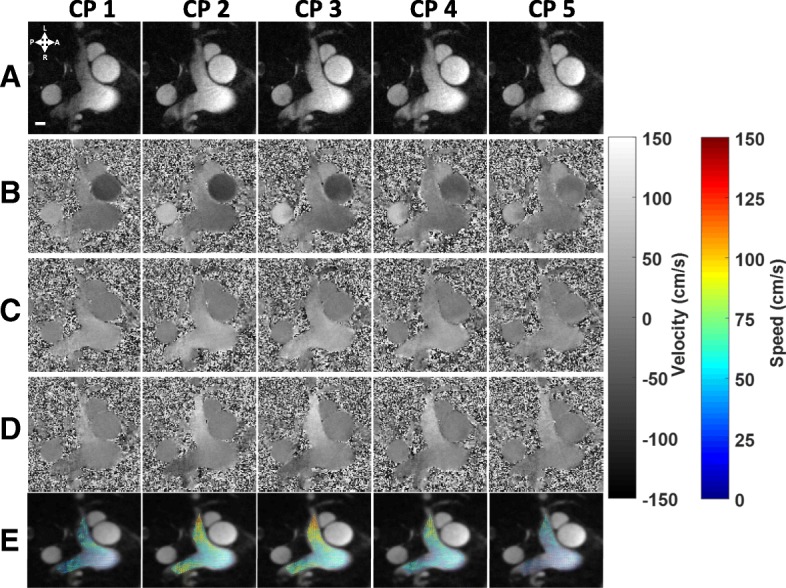
Systolic CINE frames from a healthy male adult (A3) for an axial slice at the level of the bifurcation of the main pulmonary artery (MPA) reconstructed from MOG gated radial PC-CMR acquisitions. (**a**) Magnitude images and (**b**-**d**) velocity maps corresponding to (**b**) through-plane, (**c**) anterior-posterior, and (**d**) left-right components (V_z_, V_x_, V_y_). (**e**) Vector plots of combined in-plane velocities, with color corresponding to speed (see colorbar). A white scale bar representing 10 mm is shown in the magnitude image in CP1. (Temporal resolution: 50 ms, spatial resolution: 1x1x4 mm^3^)

Representative results from the full acquisition and analysis pipeline in a healthy adult subject (A3) are presented in Fig. 3. Reconstructed magnitude and multidimensional flows are presented for five cardiac phases through systole, for an axial plane at the level of bifurcation of the pulmonary artery. These CINE frames show large in-plane and through plane flow components in the MPA while the ascending aorta (AAo) and descending aorta (DAo) contain major flow components in the through-slice direction with negligible flows in-plane. These CINE reconstructions are available as supporting Additional file 1: Video 1, while in-plane flow vectors are available as supporting Additional file 2: Video 2.

**Fig. 4:**
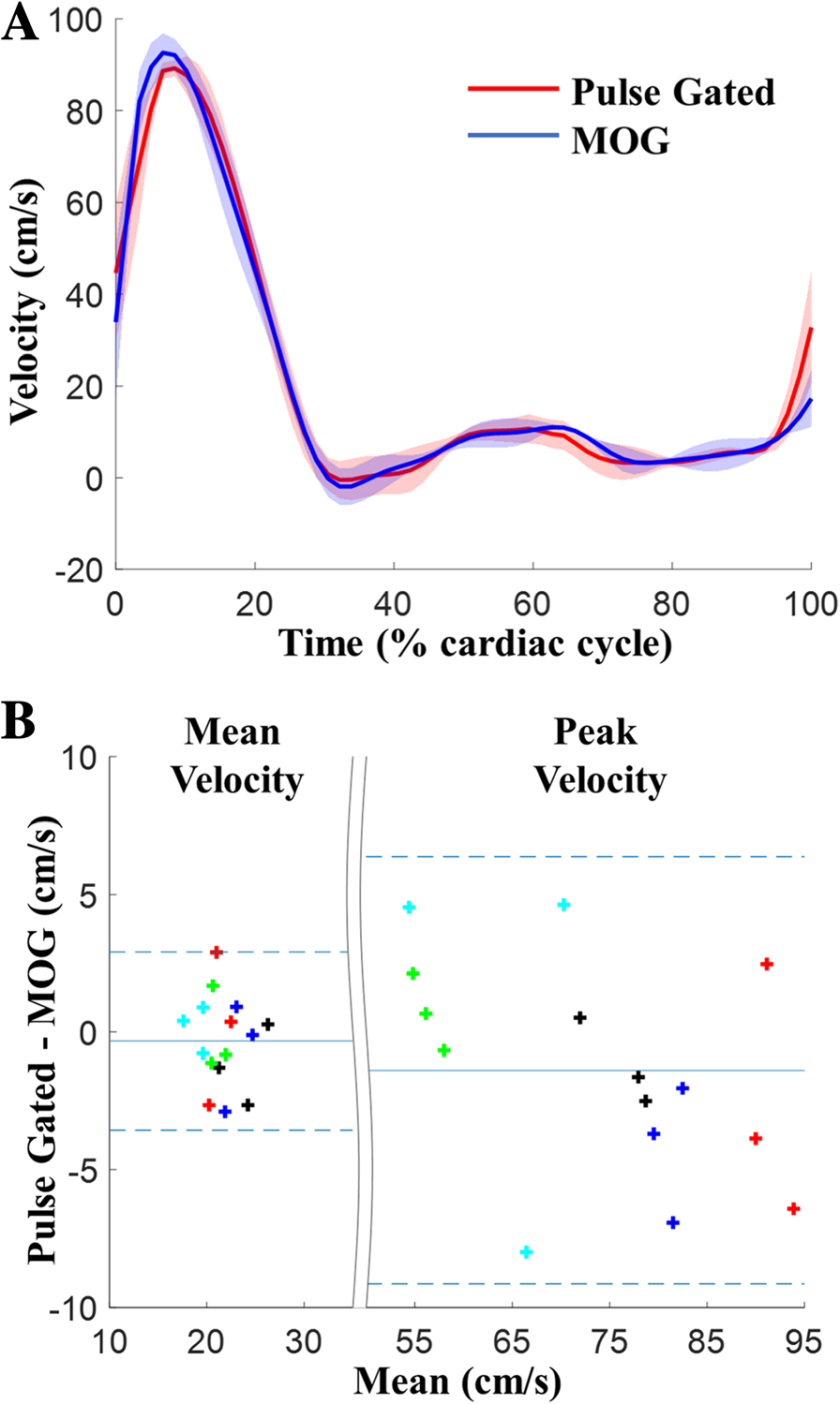
Validation of gating in healthy male adults. (**a**) Representative through-plane velocity curves from the DAo where red and blue are pulse-gated and MOG reconstructions, respectively. The shading represents the variation across the repeats in the subject. (**b**) Bland-Altman plots of the mean (bias = − 0.32 cm/s, limits of agreement [− 3.6, 2.9] cm/s) and peak (bias = − 1.4 cm/s, limits of agreement [− 9.2, 6.4] cm/s) through-plane velocities measured at the DAo from the pulse-gated and MOG CINEs are shown. The different colors in **b** represent data from the different subjects. The solid blue line represents the bias and the dotted lines show the limits of agreement

Validation of the retrospective gating approach is presented in Fig. 4. Figure 4a shows the through-plane velocity waveforms in the DAo from subject A1. There is high temporal correlation (~ 0.98) between the waveforms obtained from the two gating methods. This is evidenced by the consistency of our results with previous MOG studies [17] and the high correlation between the through-plane velocity waveforms across all subjects (Table 2). Bland-Altman plots for the mean and peak through-plane velocities obtained from the MOG and pulse-gated reconstructions are shown in Fig. 4b. There is high agreement between the measured flows from the CINEs as shown by the small errors in the mean and peak velocities. The bias and limits of agreement (two standard deviations) were − 0.32 cm/s and [− 3.6, 2.9] cm/s respectively for the mean through-plane velocities across all subjects. The corresponding measures for the peak through-plane velocities were − 1.4 cm/s and [− 9.2, 6.4] cm/s. The Wilcoxon signed rank test indicated that measurements from both gating methods were statistically indifferent with *p* = 0.60 for the mean velocities and *p* = 0.25 for the peak velocities. Movies showing representative real-time frames (temporal resolution of 26 ms) used for spatiotemporal MOG are available in supporting Additional file 3: Video 3.

**Table 2:** Table shows the MOG trigger timing error and mean Pearson correlation between pulse-gated and MOG derived flow curves for each subject

Subject	Imaging Plane	Timing Error (ms)	R_velocity waveform_
A1	MPA	17 ± 3	0.98
A2	MPA	25 ± 4	0.99
A3	MPA	19 ± 4	0.98
A4	4CH	17 ± 5	0.98
A5	4CH	22 ± 4	0.96

R: Pearson correlation coefficient; MPA: axial plane at the level of the pulmonary artery bifurcation; 4CH: four-chamber view

### Fetal motion compensation

**Fig. 5:**
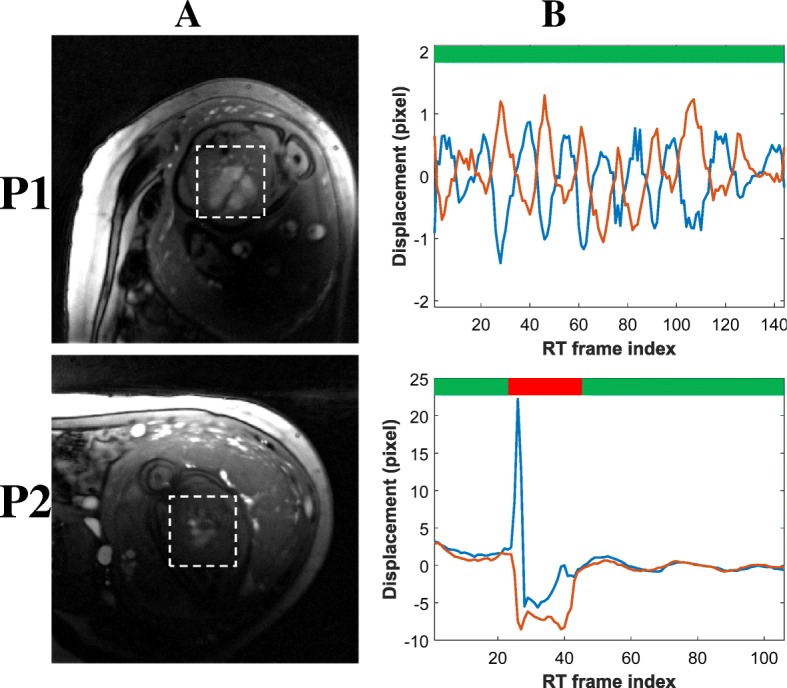
Motion curves derived using template matching translational registration for pregnant participants P1 and P2 respectively. Column **a**: time average of the real-time series, showing the ROI used for motion tracking (white dashed rectangles). Column **b**: tracked motion of the ROI (blue: horizontal, red: vertical), with the green bar identifying periods of quiescence and the red bar periods of gross-fetal motion (identified based on mutual information – see Theory)

Figure 5 depicts representative motion tracking and correction for subjects P1 and P2. The first case contained no observable gross fetal motion, whereas the second case presents spikes in the motion curve (Fig. 5b). Using the mutual-information approach described earlier, this discontinuity was detected as through-plane fetal motion (red bar in Fig. 5b). For P2, the boxplot had a median mutual information of 0.96 and a lower quartile of 0.9 with no outliers, indicating an absence of through-slice motion. For P2, the boxplot had a median of 0.96 and a lower quartile of 0.87, with 17% of the scan identified as through-plane motion based on mutual information outliers. In this case, the second quiescent period (55% of the acquisition) was used for fetal flow reconstructions. During that period, in-plane motion of the ROI was subtle, ranging between [− 0.8 1.2]_*x*_ and [− 0.9 0.7]_*y*_ pixels.

Representative real-time fetal images used for motion compensation and MOG for subject P1 are shown in supporting Additional file 4: Video 4. Although the quality of these intermediate reconstructions is not diagnostic as a result of extremely high undersampling factors (13 and 100 respectively), they are sufficient for extracting translational motion and the gating signal, as demonstrated by the final high-quality CINE output from the pipeline (final frame of V4).

### Fetal CINE PC-CMR

**Fig. 6:**
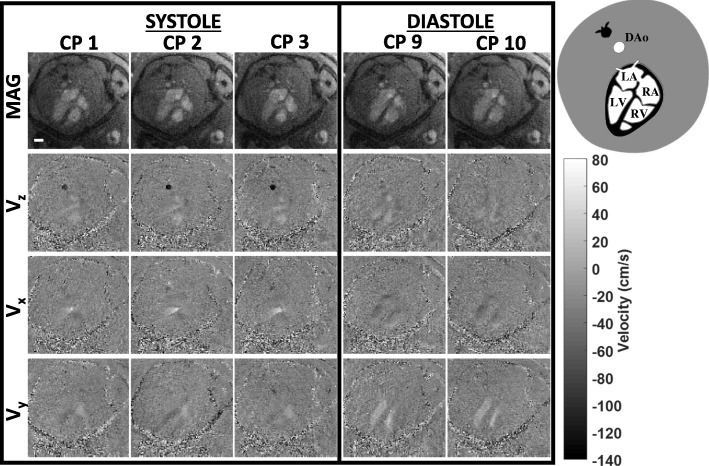
Four-chamber views from subject P1 during fetal systole and diastole. Magnitude frames are shown in the top row with velocity components below (V_z_, V_x_, V_y_). Filling of the ventricles is shown during diastole and ejection of blood from the left ventricle in the aorta is shown during systole. Labels for the fetal structures are shown in the diagram on the top right corner. A white scale bar representing 10 mm is shown in the magnitude image in CP1. (Temporal resolution: 30 ms, spatial resolution: 1x1x4 mm^3^)

**Fig. 7:**
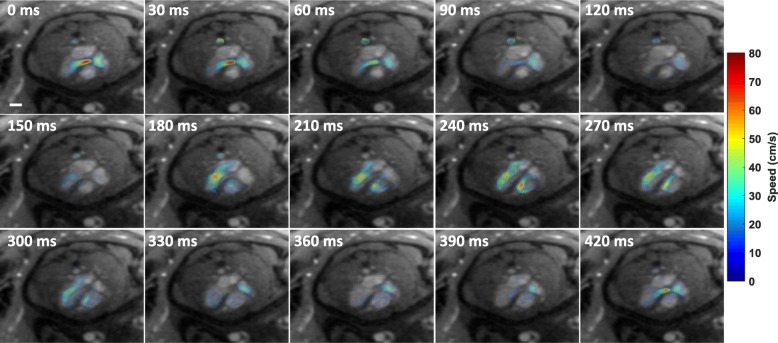
Representative visualizations of in-plane flow in a fetal four-chamber view (P1). Color-coded velocity vector for 15 cardiac phases are shown. A white scale bar representing 10 mm is shown in the image at time 0 ms. (Temporal resolution: 30 ms, spatial resolution: 1x1x4 mm^3^)

Representative reconstructions depicting complex fetal hemodynamics are presented in Fig. 6. This example provides four-chamber views through systole and diastole (subject P1). The in-plane velocity sensitive images illustrate diastolic filling of the ventricles and systolic ejection of blood from the left ventricle in the aorta. Using the in-plane velocities in the fetal heart, these dynamics are further illustrated by color-coded velocity vector and streamline plots in Fig. 7. Complex cardiac flows with vortex formations are observed in both ventricles consistent with previous fetal ultrasound findings [23]. The figure also illustrates flow vortices in the DAo, consistent with adult 4D PC-CMR studies of flow in the proximal aortic arch [24]. Furthermore, the through-plane velocity waveforms obtained from subjects P1, P4 and P5 over a cross-section of the DAo showed inter-subject consistency with an average velocity of 27.1 ± 0.4 cm/s and peak systolic velocity 70.0 ± 1.8 cm/s. The measured velocity waveforms had an ICC of 0.95. CINE reconstructions for P1 are available as supporting Additional file 5: Video 5, while in-plane flow vectors are available as supporting Additional file 6: Video 6.

**Fig. 8:**
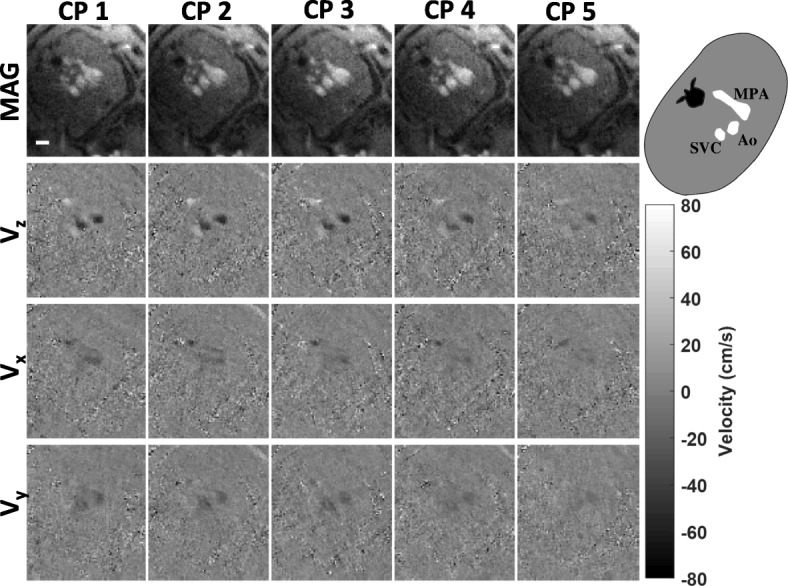
CINEs from subject P3 showing the superior vena cava, aorta and main pulmonary artery. Magnitude frames are shown in the top row with velocity components below (V_z_, V_x_, V_y_). Labels for fetal structures are shown in the diagram on the top right. A white scale bar representing 10 mm is shown in the magnitude image in CP1. (Temporal resolution: 28 ms, spatial resolution: 1x1x4 mm^3^)

Figure 8 depicts reconstructed frames from a fetal three-vessel view during systole (P3), showing flow through the superior vena cava, AAo and MPA. In this slice, the primary flow component is through-plane (V_z_). Intra-subject reproducibility of fetal measurements in P3 showed conservation of the through-plane velocity waveform obtained from the AAo (ICC = 0.97) and MPA (ICC = 0.99) in the repeated measurements. Furthermore, the pulsatility indices for the MPA were 2.5 and 2.9 and for the AAo were 2.8 and 3.1 in the two measurements, comparable to values in the literature based on ultrasound [25, 26].

## Discussion

In this study, we devised an approach for quantifying multidimensional fetal blood flow. A golden angle radial sampling scheme with multidimensional flow encoding was developed whereby both the golden-angle radial trajectory and the velocity encoding direction were continuously updated in synchrony. Reconstruction of intermediate real-time images allowed for motion correction and gating, leading to CINE reconstruction and single slice vector flow maps at high spatial and temporal resolution. Validation of the proposed sequence was performed in a flow phantom and validation of the retrospective gating and CINE reconstruction was performed in healthy adult subjects. Feasibility of the approach was tested in healthy pregnancies. Through this approach, we have generated the first multidimensional PC-CMR visualizations of complex hemodynamics in the human fetal heart.

In clinical practice, ultrasound is the primary imaging modality for fetal anatomy and flow. However, ultrasound scanning is susceptible to artifact from acoustic shadowing, or low levels of amniotic fluid (oligohydramnios), particularly in late gestation. In cases where ultrasound examinations are insufficient, Fetal CMR provides complementary information which may improve management of at-risk pregnancies. Along with improved tissue contrast, CMR can measure fetal blood flow in arbitrary planes and quantify blood oxygen saturation and hematocrit [27]. These measurements can provide a map of fetal flow and oxygen, helping us improve our understanding of how CHD affects fetal development, as well as the efficacy of CHD treatments.

A strength of the proposed approach is its ability to track motion in the real-time reconstructions and then correct for that motion in the acquired k-space data. Previous fetal PC-CMR studies have relied on lengthy Cartesian scans in which gross motion was evident only as significant artifact in the final images. When faced with obvious motion artifact, Cartesian scans are typically repeated. Our current work using radial PC-CMR demonstrated that periods of fetal through-plane motion can be identified and removed, and that residual in-plane motion from maternal breathing can be corrected. As a result, repeat measurements can be avoided, providing the vessel of interest remains in the slice plane for at least a portion of the scan.

Past implementations of fetal PC-CMR with MOG have used a simple two-parameter heart rate model. In this current work, a multiparameter MOG model was used instead, which accounts for variations in fetal heart rate throughout the acquisition. This multiparameter model was previously implemented and validated for fetal anatomical CMR [17]. Here, the gating approach was validated for PC-CMR in adults and showed high accuracy when compared with pulse-gating RR intervals (timing error ~ 20 ms). Furthermore, flow curves obtained using this approach were highly correlated with those using pulse-gating (*R* = 0.98). Finally, fetal flows obtained using this approach were consistent with those reported in the literature using through-plane Cartesian PC-CMR, and dynamic fetal cardiac structures were well visualized [9, 10]. Alternative strategies for retrospective fetal cardiac gating have recently been published using self-gating [28, 29] and iterative outlier rejection with cardiac synchronisation [30], while prospective gating using an external ultrasound probe has also been demonstrated [31].

In the proposed approach, two real-time reconstructions were performed with one at lower temporal resolution for motion correction of the acquired data and the other at higher temporal resolution for gating of the motion corrected data. Another approach would have been to perform one real-time reconstruction of the acquired data at the higher temporal resolution, and then to average frames together to improve image quality for motion correction. However, since CS is a non-linear process, such averaging is corrupted by structural noise which reduces accuracy of the registration. Another consideration would have been to combine the MOG and the CINE reconstruction steps. This would have avoided the need to reconstruct real-time images at high temporal resolution. However, this approach would have required CS in each iteration of the MOG optimization, resulting in a significant computational burden and impractical reconstruction times.

In this work, ROIs were set manually and in general, the reconstruction is robust to small deviations in ROI placement provided that the fetal chest is encompassed for motion correction and gating. An alternative approach proposed by Demesmaeker et al. 2017, uses the frequency information derived from the temporal Fourier transform of real-time reconstructions to automatically detect an ROI containing the fetal heart in anatomical images using a balanced steady state free precessionsequence [32]. Translating this approach to the PC-CMR sequence used in this work may alleviate the need for manual steps in the reconstruction, strengthening the clinical feasibility of our reconstruction pipeline.

Previous PC-CMR studies of fetal blood flow have used Cartesian sampling [9, 10], requiring relatively long scan times (~ 30 s for through-slice flows) at limited spatial resolution (1.25 × 1.25 × 5 mm^3^). The proposed radial PCMR approach provides much higher spatial resolution (1 × 1 × 4 mm^3^) and lower scan times, improving quantification and visualization of fetal flow by reducing partial volume effects. Further improvements may be achieved using more efficient sampling schemes, such as spiral PC-CMR [33]. Regardless of the trajectory that is chosen, additional applications of the approach to multi-dimensional flow proposed in this work may include uncooperative postnatal subjects such as neonates or the elderly, where motion corruption can be problematic.

Despite the success of the proposed pipeline, there are limitations with fetal PC-CMR. First, our study was performed at 3 T whereas many clinical scanners are 1.5 T, and the reduced signal-to-noise ratio (SNR) at 1.5 T could affect the accuracy of the proposed pipeline. PCMR at earlier gestation demands even higher spatial resolution because fetal structures are less developed, further affecting SNR. Finally, coil coverage over the maternal abdomen will influence image quality, depending on fetal and maternal habitus. Collectively, these limitations will require designing coils targeted for pregnancy scans and re-optimising the parameters used in CS for the lower SNR.

## Conclusion

In conclusion, we have proposed a novel radial sampling strategy for multidimensional PC-CMR. This approach provides real-time reconstructions, used to correct motion and retrospectively gate CINE reconstructions. Validation of the pipeline was performed in a flow phantom and healthy adult subjects, and its feasibility was tested in healthy pregnancies. This work resulted in the first quantification and visualisation of multidimensional fetal blood flow using PC-CMR, providing future tools for studying fetal hemodynamics, and new ways to manage patients with suspected fetal CHD.

## Additional files

Additional file 1: **Video V1.** CINE frames from a healthy male adult (A3) along an axial slice at the level of the bifurcation of the MPA reconstructed from MOG gated radial PCMR acquisitions. The video corresponds to same reconstructions shown in Fig. 3. Magnitude images and velocity maps corresponding to through-plane, anterior-posterior, and left-right components (Vz, Vx, Vy). (Temporal resolution: 50 ms, spatial resolution: 1x1x4 mm^3^). (MP4 2565 kb)

Additional file 2: **Video V2.** Vector plots of combined in-plane velocities from a healthy male adult (A3), with color corresponding to speed. The video corresponds to the same reconstruction shown in Fig. 3e. (Temporal resolution: 50 ms, spatial resolution: 1x1x4 mm^3^). (MP4 945 kb)

Additional file 3: **Video V3.** Representative real-time frames used for spatiotemporal MOG. (temporal resolution: 26 ms, spatial resolution 1x1x4 mm^3^). (MP4 6549 kb)

Additional file 4: **Video V4.** Magnitude reconstructions at different stages in the pipeline from subject P1. MOCO: representative real-time reconstruction used for motion compensation. MOG: representative real-time reconstruction used for metric optimised gating. Recombined MOG: CINE image with optimised metric obtained from reordered combination of real-time images. CINE: the final reconstruction obtained by resorting radial acquisitions based on obtained heart rate. (MP4 6730 kb)

Additional file 5: **Video V5.** Four-chamber CINE frames from subject P1 during a fetal cardiac cycle. Magnitude frames and velocity components (Vz, Vx, Vy) are shown. The video corresponds to same reconstructions shown in Fig. 6. (temporal resolution: 30 ms, spatial resolution 1x1x4 mm^3^). (MP4 1585 kb)

Additional file 6: **Video V6.** Representative visualization of in-plane flow in a fetal four-chamber view (P1) during a cardiac cycle in the form of a color-coded velocity vector. (Temporal resolution: 30 ms, spatial resolution: 1x1x4 mm^3^) (MP4 822 kb)

## Abbreviations

AAo: ascending aorta
CD: complex differences
CHD: congenital heart disease
CMR: cardiovascular magnetic resonance
CS: compressed sensing
CV: Cartesian velocity
DAo: descending aorta
ECG: electrocardiogram
GRE: gradient recalled echo
ICC: intraclass correlation coefficient
MOG: metric optimised gating
MPA: main pulmonary artery
PC-CMR: phase contrast cardiovascular magnetic resonance
R: Pearson correlation coefficient
ROI: region of interest
RV: radial velocity
SNR: signal-to-noise ratio
STV: spatial total variation
TFT: temporal Fourier transform
TR: repetition time
TTV: temporal total variation
